# The impact of prevention‐effective PrEP use on HIV incidence: a mathematical modelling study

**DOI:** 10.1002/jia2.26034

**Published:** 2022-11-17

**Authors:** D. Allen Roberts, Daniel Bridenbecker, Jessica E. Haberer, Ruanne V. Barnabas, Adam Akullian

**Affiliations:** ^1^ Department of Epidemiology University of Washington Seattle Washington USA; ^2^ Institute for Disease Modeling Bill & Melinda Gates Foundation Seattle Washington USA; ^3^ Center for Global Health Massachusetts General Hospital Boston Massachusetts USA; ^4^ Department of Medicine Harvard Medical School Boston Massachusetts USA

**Keywords:** HIV prevention, modelling, cost‐effectiveness, PrEP, Africa, adherence

## Abstract

**Introduction:**

Models that project the impact and cost‐effectiveness of HIV pre‐exposure prophylaxis (PrEP) must specify how PrEP use aligns with HIV exposure. We hypothesized that varying PrEP use according to individual‐level partnership dynamics rather than prioritization to population subgroups based on average risk will result in larger incidence reductions and greater efficiency.

**Methods:**

We used an individual‐based network transmission model calibrated to HIV dynamics in Eswatini to simulate PrEP use among individuals ages 15–34 between 2022 and 2031 under two paradigms of PrEP delivery: “Risk Group” and “Partnership.” In the “Risk Group” paradigm, we varied PrEP coverage by risk groups (low, medium and high) defined by average partnership frequency and concurrency. In the “Partnership” paradigm, all individuals are potentially eligible for PrEP, but we assumed use occurs only during partnerships and varied prioritization by partner HIV status (no prioritization to high prioritization with HIV‐positive partners). We calculated person‐time on PrEP and incidence relative to a no PrEP scenario and estimated efficiency as the person‐years of PrEP needed to avert one additional infection (NNT).

**Results:**

In the Risk Group paradigm, restricting PrEP to the high‐risk group was the most efficient (NNT = 17), but the number of infections averted was limited by the small size of the high‐risk group. Expanding PrEP use to all risk groups averted up to three times more infections but with lower efficiency (NNT = 202). PrEP use under the Partnership paradigm was 2–6 times more efficient (NNT = 33–102) than the Risk Group paradigm with all groups eligible for PrEP. A 33% reduction in incidence among 15‐ to 34‐year‐olds was achieved at 46% (95% CI: 39–52%) PrEP coverage in the Risk Group paradigm and 6% (95% CI: 5–7%) to 17% (95% CI: 14–20%) in the Partnership paradigm.

**Conclusions:**

Modelling PrEP use based on risk groups resulted in a sharp trade‐off between PrEP efficiency and impact, whereas PrEP use predicated on partnerships resulted in much higher efficiency for widespread PrEP availability. Model estimates of PrEP impact and cost‐effectiveness in generalized epidemics are strongly influenced by assumptions about how PrEP use aligns with individual‐level HIV exposure heterogeneity.

## 1 INTRODUCTION

HIV incidence remains high in sub‐Saharan Africa, with an estimated 870,000 people newly inflected in 2020 [1]. Daily oral tenofovir disoproxil fumarate in combination with emtricitabine as HIV pre‐exposure prophylaxis (PrEP) is a safe and effective prevention strategy, and early implementation has begun in sub‐Saharan Africa [2, 3, 4]. Policymakers must decide how to allocate finite HIV budgets to PrEP programmes versus other interventions to maximize the impact on HIV incidence and mortality. To guide policy, mathematical modelling analyses have estimated that PrEP may be cost‐effective if prioritized to subgroups with higher risk (e.g. female sex workers). However, widespread PrEP use in generalized epidemics is seen as prohibitively expensive, because the eligible population is large and the average incidence rate is lower [5, 6]. These results have influenced World Health Organization (WHO) guidelines that recommend PrEP for populations with an average HIV incidence of at least 3 per 100 person‐years (PY), as well as national guidelines that specify risk criteria for PrEP eligibility [7].

To estimate the cost‐effectiveness of PrEP use among subgroups with different levels of risk, mathematical models must specify heterogeneity in HIV exposure. A common approach is to stratify the simulated population into a few broadly defined “risk group” categories with different average levels of partnership turnover, mixing patterns, sexual frequency and condom use [8, 9]. As a result, strata (e.g. age/gender/risk group) with unique sets of sexual activity parameters have different HIV incidence rates, but all individuals within a given stratum are subject to the same average incidence rate. This specification is common in compartmental models because it introduces some HIV exposure heterogeneity without needing to explicitly represent individuals in computer memory.

Despite its convenience, the risk group approach imposes restrictions for modelling PrEP. HIV exposure is highly dynamic depending on an individual's current number and types of partnerships, partner HIV status or use of other preventive interventions. In the “prevention‐effective” paradigm, these time‐varying behaviours also influence individual decisions to start and stop PrEP [10]. Individual partnerships are not explicitly simulated in the risk group approach, removing a component of heterogeneity that may be important for modelling PrEP. If, within risk groups, PrEP use is higher among individuals with a higher probability of HIV exposure, then models ignoring this heterogeneity may overestimate the cost and underestimate the impact of PrEP use. Individual‐based models that allow PrEP use to be contingent on dynamic partnership characteristics have been used for men who have sex with men in the United States [11] but rarely in generalized epidemics in sub‐Saharan Africa.

Here, we use an individual‐based model previously calibrated to the HIV epidemic in Eswatini to evaluate the effect of aligning PrEP use with HIV exposure on estimates of the impact and efficiency of PrEP scale‐up in young men and women. We compared models in which PrEP coverage varies by risk group to models in which PrEP use occurs only while individuals are in partnerships and varies by partner HIV status. We estimated HIV incidence and person‐years on PrEP for each PrEP implementation paradigm at increasing levels of PrEP coverage and compared the projected relationship between additional PrEP use and impact across model assumptions.

## 2 METHODS

### 2.1 Model description

We used Epidemiological MODelling Software (EMOD), an open‐source, stochastic individual‐based model that simulates demography, disease progression, partnership networks and intervention use among a collection of individuals [12]. For this analysis, we used a model recently calibrated to transmission dynamics in Eswatini, which has been described in detail previously [13]. This model provided a convenient environment for comparing multiple approaches for simulating PrEP delivery in a high‐incidence setting where PrEP implementation is underway. Individuals in this model are stratified into three risk groups with different sexual activity parameters: a small “high‐risk” group intended to represent sex workers and clients, a “medium‐risk” group with short‐term partnerships and a large “low‐risk” group with fewer, longer‐term partnerships. Additional details on risk group sizes, incidence rates and partnership dynamics are provided in Supplementary Material 1 (Tables S1.1–S1.3). HIV transmission occurs through heterosexual partnerships formed between individuals, which are governed by age, sex and risk group‐specific pair formation parameters [14, 15]. Partnerships formed between individuals are stratified by types with specific duration distributions, condom use probabilities and concurrency propensity. Marital partnerships are of longer duration and involve older individuals, while informal and transitory partnerships are shorter and tend to involve younger individuals. Commercial partnerships are the shortest and can be formed between individuals in the highest‐risk group, with high turnover and concurrency. Among 15‐ to 34‐year‐olds, on average 35% of men and 52% of women in the model have at least one partner at a given moment in time, similar to observed data from cohorts in southern Africa [16, 17]. Partnership dynamics in EMOD also recapitulate age‐specific transmission patterns observed in cohort studies conducted in KwaZulu‐Natal, South Africa [18, 19, 20]. The model was calibrated using a parallel simultaneous optimization algorithm, and 250 sets of 24 fitted parameters were selected using roulette resampling in proportion to the likelihood to capture stochastic and parameter uncertainty [21]. We assume that antiretroviral therapy (ART) coverage reached UNAIDS 90‐90‐90 targets by 2020 and voluntary medical male circumcision (VMMC) coverage remains constant at 2016 levels [22]. Transmission from individuals on ART is assumed to be reduced by 92% per the coital act [23]. Further details on the model parameters and calibration are available in Supplementary Material 2 (Table S2.1 and Figures S2.1 and S2.2).

### 2.2 PrEP paradigms and scenarios

**Figure 1:**
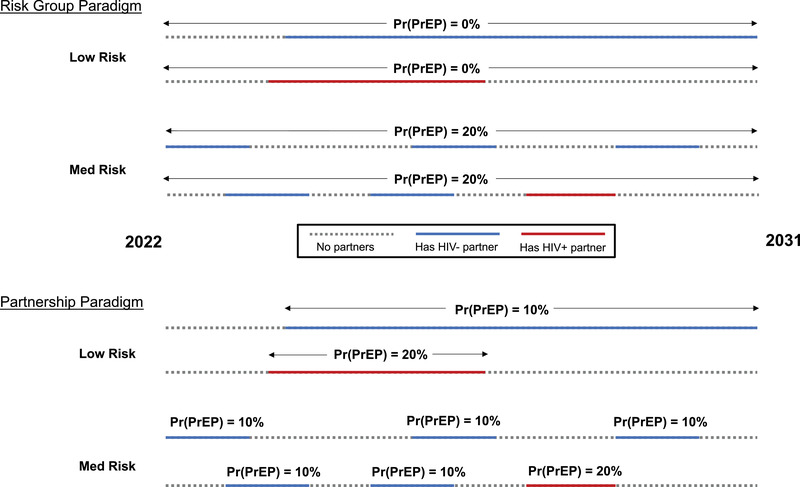
Illustration of PrEP use assumptions according to two model paradigms. Four example individual trajectories over a ten‐year period are shown in each paradigm. In the Risk Group paradigm, PrEP coverage can vary according to behavioural risk group, but PrEP use within risk group is independent of partnership status. In the Partnership paradigm, PrEP use occurs only during partnerships and may be prioritized with HIV‐positive partners. In this example, PrEP coverage is twice as high during partnerships with HIV‐positive individuals compared to during partnerships with HIV‐negative individuals. The high‐risk group is not shown. PrEP coverage levels are systematically varied in the main analysis. Abbreviation: Pr(PrEP), PrEP coverage.

We focused this analysis on individuals ages 15–34 and restricted our analyses to a 10‐year period from 2022 through 2031. We simulated PrEP use among 15‐ to 34‐year‐olds according to two paradigms (Figure 1). In the “Risk Group” approach, PrEP coverage can vary by risk group, but PrEP use within the risk group is independent of partnership status. In the “Partnership” approach, low‐ and medium‐risk individuals are assumed to only use PrEP while in a partnership. Furthermore, because reporting a partner with HIV or a partner of unknown status is a strong predictor of PrEP use [24, 25, 26, 27], we allow partner HIV status to modify the probability of PrEP use. Specifically, we applied a multiplier for PrEP coverage among individuals with HIV‐positive partners relative to those with only HIV‐negative partners. We varied this multiplier from 1 (partner HIV status has no impact on PrEP coverage) to 5 (individuals with an HIV‐positive partner are five times more likely to use PrEP than individuals with only HIV‐negative partners) (Table 1), reflecting uncertainty in the relationship between actual HIV partner status and PrEP use. These multipliers lead to different levels of prioritization of PrEP use during periods of HIV exposure (SM1, Figure S1.1). In sensitivity analyses, we apply this multiplier only to individuals in partnerships with HIV‐positive individuals who have been diagnosed. Based on our model parameterization, high‐risk individuals are nearly always in partnerships and cycle in and out of short‐term commercial partnerships on a faster time scale than PrEP refill schedules (typically 1–3 months), making the Partnership paradigm infeasible for the high‐risk group. Instead, we assigned high‐risk individuals the same PrEP coverage as low‐ and medium‐risk individuals with an HIV‐positive partner. Therefore, the Partnership paradigm differs from the Risk Group paradigm primarily in how low‐ and medium‐risk individuals (∼95% of the population) are assumed to use PrEP. For each scenario, we systematically vary PrEP coverage from low levels up to a maximum 90% coverage in any subgroup (SM1, Tables S1.4 and S1.5). In all scenarios, PrEP use occurs in month‐long intervals according to the timestep of the model; initiation and discontinuation on shorter time scales (e.g. “on‐demand PrEP”) is not modelled. Therefore, a given individual could have at most six PrEP initiations in a year. We assumed that PrEP confers a 75% reduction in HIV acquisition risk per coital act [28]. We simulated each PrEP coverage level under each PrEP scenario, as well as a scenario without PrEP, for each of the 250 parameter sets.

**Table 1:** Model assumptions for PrEP use according to the Risk Group and Partnership paradigms

Paradigm	Risk Group	Partnership requirement	Multiplier for PrEP use with HIV‐positive partners relative to PrEP use with HIV‐negative partners
Risk Group	All	No	–
Risk Group	High and medium	No	–
Risk Group	High	No	–
Partnership	All	Yes	1
Partnership	All	Yes	2
Partnership	All	Yes	3
Partnership	All	Yes	5

Note: In the Risk Group paradigm, PrEP use can differ according to risk group, but within risk group, PrEP use is assumed to be independent of partnerships and partner characteristics. In the Partnership paradigms, PrEP use is assumed to only occur during partnerships, and a multiplier is applied to PrEP coverage among individuals with one or more HIV‐positive partners relative to PrEP coverage among individuals with only HIV‐negative partners.

### 2.3 Analysis

We directly compared estimates of PrEP impact and efficiency between the Partnership and the Risk Group approaches as follows. For each PrEP simulation, we calculated total person‐time on PrEP, PrEP coverage among 15‐ to 34‐year‐olds, the percentage of infections averted relative to a scenario without PrEP and the relative risk (RR) of infection among 15‐ to 34‐year‐olds compared to a scenario without PrEP (Table 2). As a proxy for cost‐effectiveness, we estimated the relationship between person‐time on PrEP (cost) and infections averted (impact) for each PrEP scenario by fitting a one‐knot natural spline regression model with a random intercept on parameter set (SM1, Figure S1.2). We used an analogous model to describe the relationship between PrEP coverage and RR among 15‐ to 34‐year‐olds. We calculated the additional person‐years of PrEP needed to avert one infection (number needed to treat, NNT) as the average slope between infections averted and person‐years on PrEP (SM1, Figure S1.3). We summarized parameter and stochastic uncertainty by calculating 95% credible intervals (CI) for each metric. We additionally conducted stratified analyses by gender. All statistical analysis was conducted in R version 4.0.2 [29]. The code used to run EMOD for this analysis is available on GitHub (https://github.com/dallenroberts/prep‐risk).

**Table 2:** Metrics calculated for each PrEP simulation

Metric	Definition	Interpretation
Person‐time on PrEP	Total person‐years of PrEP use from 2022 to 2031	Programme cost
PrEP coverage	Proportion of HIV‐negative 15‐ to 34‐year‐olds currently on PrEP, averaged from 2022 to 2031	Reach among potential users
% of infections averted	Percentage of total infections (all age groups) occurring between 2022 and 2031 in a simulation with no PrEP that are averted in a given PrEP simulation	Total impact
Relative risk (RR)	Average HIV incidence rate among all 15‐ to 34‐year‐olds (regardless of PrEP use) between 2022 and 2031 in a given PrEP simulation divided by the same incidence rate in a simulation with no PrEP	Impact among potential users
Number needed to treat (NNT)	Average additional person‐years of PrEP use in a given PrEP simulation needed to avert one additional HIV infection between 2022 and 2031 relative to a simulation with no PrEP	Efficiency or cost‐effectiveness

Note: Each metric represents a different construct (e.g. impact and cost) through which PrEP programmes are evaluated.

## 3 RESULTS

### 3.1 PrEP scenario results

**Figure 2:**
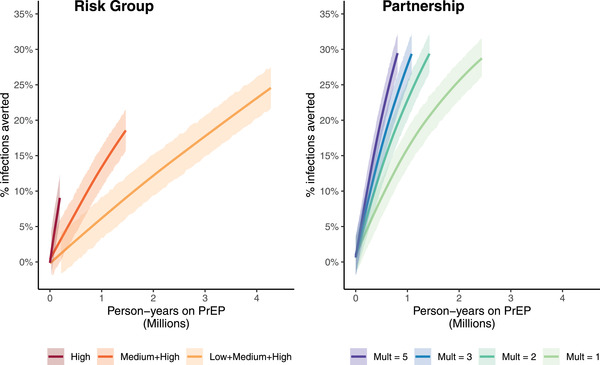
Relationship between additional person‐time on PrEP and percentage of infections averted relative to a no PrEP scenario, by PrEP paradigm. Person‐years on PrEP and percentage of infections averted are cumulative across a 10‐year period spanning 2022–2031. Shaded regions indicate 95% credible intervals. Abbreviation: Mult, multiplier on the probability that an individual with an HIV‐positive partner will use PrEP relative to the probability that an individual with only HIV‐negative partners will use PrEP.

The relationship between additional person‐time on PrEP and infections averted depended strongly on the PrEP scenario assumed (Figure 2). In the Risk Group scenarios, prioritizing PrEP to the high‐risk group was most efficient (NNT = 17, Table 3), but the percentage of infections averted at the highest PrEP coverage modelled (90% of high‐risk individuals) was limited to 9% (95% CI: 6–12%). The percentage of infections averted at 90% coverage was higher with PrEP uptake among other risk groups (high and medium: 19% [95% CI: 16–21%]; all: 25% [95% CI: 22–27%]), but the efficiency was lower (high and medium: NNT = 86; all: NNT = 202). In the Partnership paradigm, the efficiency depended on the multiplier governing the prioritization of PrEP during periods of higher risk (multiplier = 1: NNT = 102; multiplier = 5: NNT = 33). The Partnership scenarios were 2–6 times more efficient than the Risk Group scenario with all individuals eligible for PrEP. However, the total impact of the Partnership scenarios (>25% of infections averted at the highest coverage modelled in all scenarios) was not limited by the size of the risk groups, since all individuals are potentially eligible for PrEP. The contrast between the Risk Group and Partnership paradigms was similar when stratified by gender (SM1, Figure S1.4 and Table S1.6).

**Table 3:** Number of additional person‐years of PrEP needed to avert one additional HIV infection (number needed to treat, NNT), by PrEP scenario

Paradigm	Scenario	NNT (95% CI)
Risk Group	All	202 (200–204)
	High and medium	86 (85–87)
	High	17 (17–18)
Partnership	Mult = 1	102 (101–103)
	Mult = 2	58 (57–58)
	Mult = 3	45 (44–45)
	Mult = 5	33 (32–33)

Abbreviations: CI, credible interval; Mult, multiplier on the probability that an individual with an HIV‐positive partner will use PrEP relative to the probability that an individual with only HIV‐negative partners will use PrEP.

**Figure 3:**
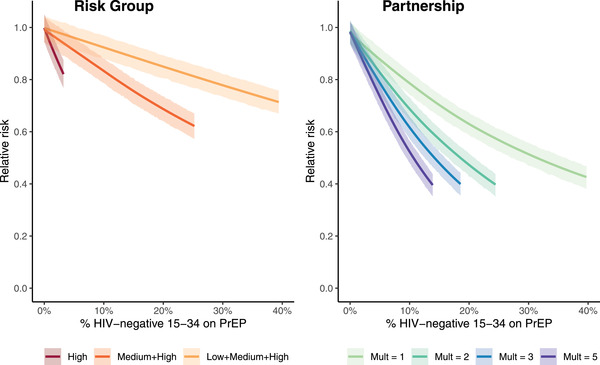
Relationship between PrEP coverage among all HIV‐negative 15‐ to 34‐year‐olds and the relative risk of HIV infection compared to a no PrEP scenario, by PrEP paradigm. Relative risk is averaged across 2022–2031. Shaded regions indicate 95% credible intervals. Abbreviation: Mult, multiplier on the probability that an individual with an HIV‐positive partner will use PrEP relative to the probability than an individual with only HIV‐negative partners will use PrEP.

The incidence reduction among 15‐ to 34‐year‐olds at given PrEP coverage levels also depended strongly on the assumed PrEP scenario (Figure 3). To achieve an RR of 0.67 (a 33% reduction in incidence relative to a no PrEP scenario) in the Risk Group scenario with equal coverage across risk groups, PrEP coverage needed to reach 46% (95% CI: 39–52%). In the Partnership scenarios, the same incidence reduction was achieved at 17% coverage (95% CI: 14–20%) with a multiplier of 1 and 6% coverage (95% CI: 5–7%) with a multiplier of 5. At 10% coverage, the impact on incidence was smaller in the Risk Group scenarios (high and medium: RR = 0.83 [95% CI: 0.79–0.88]; all: RR = 0.92 [95% CI: 0.88–0.97]) than in the Partnership scenarios (multiplier = 1: RR = 0.78 [95% CI: 0.74–0.83]; multiplier = 5: RR = 0.52 [95% CI: 0.48–0.57]). In the Risk Group scenario in which PrEP is restricted to the high‐risk group, the largest impact on incidence (RR = 0.82, 95% CI: 0.77–0.87) occurred at 90% coverage among high‐risk HIV‐negative individuals, which corresponded to 3% coverage among HIV‐negative 15‐ to 34‐year‐olds. Similar patterns were observed when stratifying by gender (SM1, Figure S1.5). In the Partnership scenario, incidence reductions were larger among men than among women at the same level of coverage. A 50% reduction in the incidence (RR = 0.5) was achieved at 9% (multiplier = 5; 95% CI: 8–11%) to 21% (multiplier = 1; 95% CI: 17–25%) coverage among men and 11% (multiplier = 5; 95% CI: 10–13%) to 41% (multiplier = 1; 95% CI: 35–46%) among women.

The distribution of averted infections by risk group also depended on the assumed PrEP scenario (SM1, Figure S1.6). In the Risk Group scenario in which all individuals were eligible for PrEP, 41% of the infections averted were among low‐risk individuals and 41% among medium‐risk individuals. In contrast, restricting PrEP to either the medium‐ and high‐risk groups or just the high‐risk group primarily averted infections among high‐ and medium‐risk individuals, with only 8–13% of averted infections among low‐risk individuals. In the Partnership scenarios, PrEP use primarily averted infections among low‐ (39%) and medium‐risk (42%) individuals, similar to the Risk Group scenario in which all individuals were eligible for PrEP.

### 3.2 Sensitivity analysis

The results from the Partnership scenarios were similar when we restricted the multiplier on PrEP coverage among individuals with HIV‐positive partners to only be applied when the partner had previously been diagnosed (SM1, Figure S1.7). Since over 90% of HIV‐positive individuals are assumed to know their status by 2020, changing this assumption had little impact on the distribution of PrEP person‐time (SM1, Figure S1.8).

## 4 DISCUSSION

Our results indicate that model projections of the impact and efficiency of PrEP programmes are highly sensitive to how closely PrEP use is assumed to align with HIV exposure. Compared to a model that ignores partnerships, PrEP use among the general population is projected to be two times more efficient if PrEP use is prioritized during partnerships and over six times more efficient if use is further prioritized among individuals with HIV‐positive partners. In addition, large incidence reductions can be achieved at low levels of PrEP coverage if PrEP use in the general population is concentrated when HIV exposure is more likely, but high levels of PrEP coverage are needed if time‐varying individual risk is ignored.

The differences in the results from the Risk Group and Partnership paradigms are driven by the low‐ and medium‐risk groups, which represent most of the population but collectively have lower average incidence rates (0.29/100 PY for men and 1.04/100 PY for women). These average incidence rates determine the efficiency of PrEP use in the Risk Group paradigm, whereas the Partnership paradigm prioritizes PrEP use during times of higher HIV risk by further considering whether the individual has a partner and the partner's HIV status. This model specification allows individuals to cycle on and off PrEP in accordance with their time‐varying HIV risk.

Estimates of the cost‐effectiveness of PrEP use in sub‐Saharan Africa will depend on the assumed alignment of PrEP use and HIV risk. While most prior models of PrEP use in sub‐Saharan Africa have used risk groups to reflect HIV exposure heterogeneity, a recent analysis from South Africa used an individual‐based model to estimate the cost‐effectiveness of PrEP use concentrated during periods of condomless sex [30]. Under this paradigm, PrEP use among 15‐ to 64‐year‐olds was cost‐effective across all simulations, whereas simulations that assumed that PrEP use was unrelated to condomless sex were never cost‐effective. As with the Partnership paradigm in our analysis, their model allowed time‐varying individual HIV risk to influence PrEP use. Our model differs by explicitly representing a network of partnerships formed between individuals, allowing partner HIV status to impact PrEP coverage. Taken together, our results indicate that PrEP may be more cost‐effective than previously estimated if use aligns with potential HIV exposure.

Evidence from PrEP implementation studies suggests that the Partnership paradigm may better reflect patterns of PrEP use compared to the Risk Group paradigm. PrEP uptake, continuation and adherence are higher among clients who report a partner with known HIV‐positive status or of unknown status [24, 25, 26, 27] or who have higher predicted risk using validated risk‐scoring tools [31, 32]. Evidence from serodiscordant couples in Kenya and Uganda also supports that PrEP adherence is more likely when the HIV transmission risk is higher [33]. Common reasons for PrEP discontinuation include low perceived HIV risk or reporting a known HIV‐negative partner [26, 27, 34, 35, 36, 37]. Furthermore, HIV incidence in many PrEP implementation studies has been substantially lower than expected in the absence of PrEP despite a minority of participants adhering to PrEP. HIV incidence among all participants was 55% lower after PrEP became available during the Evidence for Contraceptive Options in HIV Outcomes (ECHO) trial in South Africa despite only 26% of participants reporting PrEP use [38]. In a study of young women in South Africa and Zimbabwe, only 21% of participants had high adherence (≥ 700 fmol/punch by dried blood spot) at 6 months after initiation. However, adherence was significantly associated with the report of HIV risk factors [39], and the observed incidence (1.0/100 PY) was 73% lower than expected based on a modelled counterfactual [40, 41]. Our modelling results suggest that such incidence reductions at low coverage levels are possible when PrEP use is concentrated during times of higher HIV risk.

To achieve the impact estimated in the Partnership scenarios, individuals must be able to start and stop PrEP in accordance with HIV risk. Therefore, PrEP must be widely available and easily accessible. Increased distance to PrEP services is a barrier to use [42, 43], and PrEP delivery at community locations (e.g. pharmacies) may facilitate access [44]. Increased awareness and normalization of PrEP use, as well as new formulations that may mitigate adherence challenges, may increase uptake among those who need it [45, 46]. Risk perception and/or desire for HIV prevention must align with underlying HIV risk, and programmes can offer person‐centric education and counselling such that PrEP is perceived as a means to achieving sexual health and relationship goals [47, 48]. Partner HIV testing, including the secondary distribution of HIV self‐testing kits, can also improve the prioritization of PrEP use during times of HIV exposure [49]. Furthermore, the high rates of discontinuation and re‐initiation that have been observed in PrEP implementation studies [50] may in part reflect episodic use in accordance with risk rather than low adherence. Further research is needed to characterize PrEP use patterns, which can in turn inform modelling analyses. Programmes should adopt a prevention‐effective adherence lens by taking into account time‐varying individual risk factors and reasons for initiation or discontinuation when measuring PrEP coverage and persistence [51, 52]. In contrast, the Risk Group scenarios imply that a small sub‐population of individuals should be prioritized for PrEP use. Several risk‐scoring tools have been developed to identify high‐incidence subgroups based on individual‐level and geographic factors [53, 54, 55], which may allow for more targeted PrEP delivery programmes than the three risk groups included in our model and others. However, the predictive performance of these tools has been moderate, and even if such a subgroup can be identified and engaged in PrEP services, the population‐level impact is projected to be much smaller than if PrEP is used more generally due to the relatively smaller size of the high‐risk population. Furthermore, policies that associate PrEP with specific key populations may introduce stigma [56].

Our results have several limitations. First, we chose a model previously calibrated to Eswatini that allowed comparative modelling of PrEP use based on risk groups versus individual partnerships across a wide range of scenarios in a high HIV incidence setting. As such, our results do not correspond to a specific PrEP delivery programme in Eswatini. Updated calibrations with more recent data (e.g. scale‐up of ART and VMMC coverage) would be needed to make specific projections. In addition, programme costs will depend on the platform used to provide PrEP services [57]; for these reasons, we did not produce formal cost‐effectiveness estimates. Second, projections will also depend on epidemic context, and our results may not generalize to low prevalence settings where transmission primarily occurs within small sub‐populations. Nevertheless, modelling the time‐varying relationship between PrEP use and HIV exposure at the individual level should improve estimation in all settings. Third, we did not explicitly model knowledge of partner HIV status; instead, we allowed the true HIV status of partners to affect PrEP uptake. Knowledge of partner HIV status is low in southern Africa [58], so individuals considering PrEP often make decisions with considerable uncertainty about their HIV risk. However, quantitative and qualitative evidence supports that beliefs about partner status, even in the absence of disclosure, are strong determinants of PrEP use [24, 25, 26, 27, 34, 35]. For this reason, we varied the multiplier for PrEP uptake by partner HIV status across a wide range, including scenarios in which partner HIV status had no impact on PrEP use. Further research is needed to understand the degree to which decisions about PrEP use align with HIV exposure and to incorporate them into mathematical models. Fourth, the Partnership paradigm does not capture many of the reasons that individuals may choose to start or stop PrEP. Within partnerships, PrEP use is assumed to be independent of condom use and partner viral suppression. Our model may underestimate efficiency and impact if PrEP is preferentially used during periods of condomless sex or with partners with unsuppressed viral loads. Additionally, pill burden, side effects, travel times, stigma or fear of intimate partner violence act as barriers to PrEP use [24, 25, 35, 36, 59], which may limit the prioritization of PrEP use with HIV exposure. Finally, our model also does not include HIV drug resistance; however, prior analyses have estimated the limited impact of drug resistance on PrEP cost‐effectiveness estimates when dolutegravir is used in first‐line ART regimens [30].

## 5 CONCLUSIONS

Model assumptions that govern the alignment of PrEP use with HIV exposure strongly influence projections of PrEP impact. Prioritizing PrEP use based on risk groups results in a substantial trade‐off between infections averted and efficiency and may underestimate cost‐effectiveness. Assuming instead that PrEP use is prioritized based on partnership characteristics results in much higher efficiency for use in the general population. Individual‐based models may allow a better representation of the dynamics of PrEP use in relation to time‐varying individual‐level heterogeneity in HIV exposure to improve estimates of the cost‐effectiveness of PrEP programmes.

## COMPETING INTERESTS

RVB reports grants from the Bill & Melinda Gates Foundation (BMGF) and the National Institutes of Health (NIH); and Regeneron Pharmaceuticals for conference abstract and manuscript writing support, outside the submitted work. JEH reports grants from the National Institutes of Health (NIH); and consultative fees from Merck outside the submitted work. No other authors report competing interests.

## AUTHORS’ CONTRIBUTIONS

DAR, AA and RVB designed the research study. DAR and DB performed the modelling analyses. DAR, AA, JEH and RVB provided critical interpretation of results. DAR wrote the first draft of the paper. All authors have read and approved the final manuscript.

## FUNDING

DAR received support from the US National Institute of Health (NIH) [F30MH122300]. JEH received support from the US National Institute of Health [K24MH114732]. This work is based on research funded in part by the Bill & Melinda Gates Foundation, including models and data analysis performed by the Institute for Disease Modeling at the Bill & Melinda Gates Foundation.

## DISCLAIMER

The content is solely the responsibility of the authors and does not necessarily represent the official views of the National Institute of Mental Health or the National Institutes of Health.

## Supporting information


**Supplementary File 1**: Additional details on risk group sizes, incidence rates and partnership dynamics.

**Supplementary File 2**: Model parameters and calibration details.

## Data Availability

Data sharing is not applicable to this article as no datasets were generated or analysed during the current study.
