# Supplementary material for: The impact of prevention‐effective PrEP use on HIV incidence: a mathematical modelling study
Source: J Int AIDS Soc. 2022 Nov 17;25(11):e26034. doi: 10.1002/jia2.26034 (PMC9670193; doi:10.1002/jia2.26034)

Table S1.1: Population fraction and incidence rates among HIV-negative 15-34-year-old men and women in the model in 2022, stratified by risk group. CI = credible interval.

	Men		Women	
Risk group	Population fraction	Incidence per 100 PY (95% CI)	Population fraction	Incidence per 100 PY (95% CI)
Low	0.66	0.26 (0.15-0.39)	0.72	0.84 (0.55-1.15)
Medium	0.26	0.59 (0.32-0.85)	0.27	2.29 (1.64-2.92)
High	0.08	2.70 (1.70-4.09)	0.01	12.12 (5.24-22.82)

Table S1.2: Proportion of person-time spent in at least one partnership among HIV-negative 15-34-year-olds between 2022 and 2031, stratified by risk group. Parentheses indicate 95% credible intervals.

Risk Group	Men	Women
Low	0.33 (0.28-0.37)	0.48 (0.42-0.53)
Medium	0.36 (0.32-0.39)	0.55 (0.50-0.58)
High	0.85 (0.79-0.88)	1 (1-1)

Table S1.3: Proportion of partnership-time in which at least one partner was HIV-positive among HIV-negative 15-34-year-olds between 2022 and 2031, stratified by risk group. Parentheses indicate 95% credible intervals.

Risk Group	Men	Women
Low	0.08 (0.06-0.10)	0.07 (0.06-0.08)
Medium	0.13 (0.10-0.15)	0.12 (0.10-0.13)
High	0.24 (0.20-0.27)	0.39 (0.35-0.43)

Figure S1.1: In the Partnership scenario, prioritization of PrEP use during periods of HIV exposure is governed by a multiplier relating PrEP coverage among individuals with HIV-positive partners relative to those with only HIV-negative partners. As the multiplier increases, PrEP is increasingly used when a low- or medium-risk individual has an HIV-positive partner (turquoise bar), while use during HIV-negative partnerships (purple bar) decreases. Since high-risk individuals cycle in and out of very short commercial partnerships, we applied the same level of PrEP coverage as among low- and medium-risk individuals with an HIV-positive partner. Therefore, PrEP use among high-risk individuals (yellow bars) also is prioritized as the multiplier increases. Error bars indicate 95% credible intervals across 250 parameter sets.

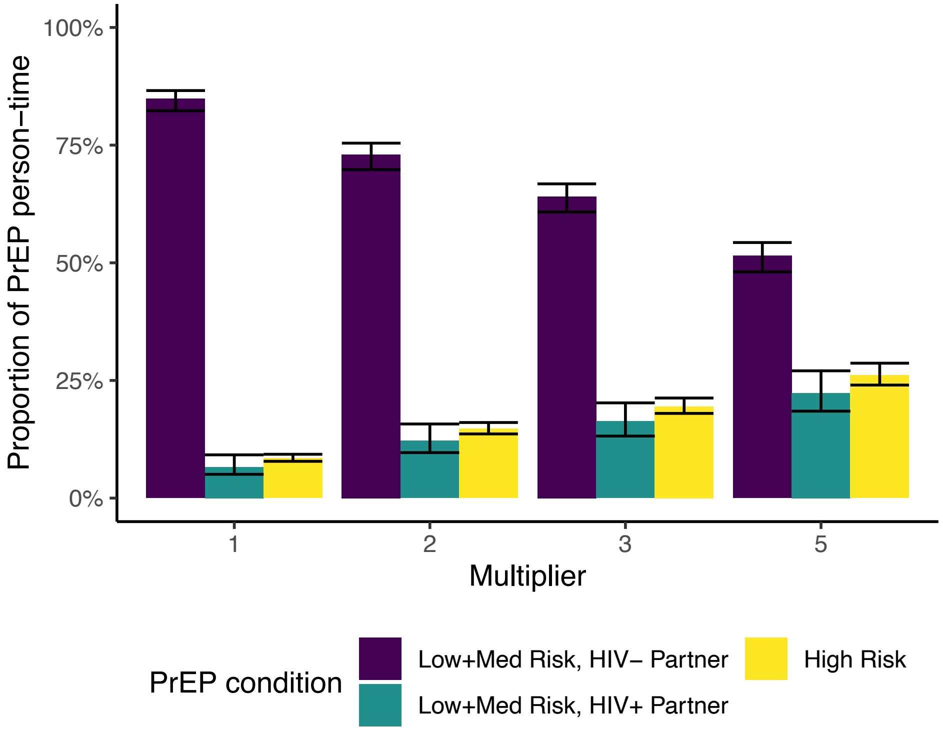

Table S1.4: Simulated PrEP coverage levels for scenarios under the Risk Group paradigm. Coverage was scaled from the lowest level to the highest level in even increments. Each coverage level was simulated for each of 250 parameter sets to capture uncertainty.

		PrEP coverage		
Scenario	Scale	Low Risk	Medium Risk	High Risk
High	Lowest	0	0	10%
High	Highest	0	0	90%
Medium + High	Lowest	0	10%	10%
Medium + High	Highest	0	90%	90%
Low + Medium + High	Lowest	10%	10%	10%
Low + Medium + High	Highest	90%	90%	90%

Table S1.5: Simulated PrEP coverage levels for scenarios under the Partnership paradigm. Multiplier indicates the multiplier for PrEP coverage level for individuals with at least one HIV-positive partner relative to PrEP coverage for individuals with only HIV-negative partners. The same multiplier was applied to PrEP coverage among high-risk individuals. Coverage was scaled from the lowest level to the highest level in even increments. Each coverage level was simulated for each of 250 parameter sets to capture uncertainty.

		PrEP coverage		
Multiplier	Scale	All HIV- partners	≥1 HIV+ partner	High Risk
1	Lowest	10%	10%	10%
1	Highest	90%	90%	90%
2	Lowest	5%	10%	10%
2	Highest	45%	90%	90%
3	Lowest	5%	15%	15%
3	Highest	30%	90%	90%
5	Lowest	3%	15%	15%
5	Highest	18%	90%	90%

Figure S1.2: Estimation of the relationship between additional PrEP use (x-axis) and percentage of infections averted relative to a scenario without PrEP (y-axis). Risk Group scenarios are on the top row while the Partnership scenarios are on the bottom row. Each black line represents one of 250 parameter sets. X-axis and y-axis scales vary for each plot to show model fit. The parameter set lines connect simulations run at various PrEP coverage levels (**Tables S1.4** and **S1.5**). The red lines show the fit of a mixed-effects regression model using a natural spline with one knot and a random intercept for parameter set.

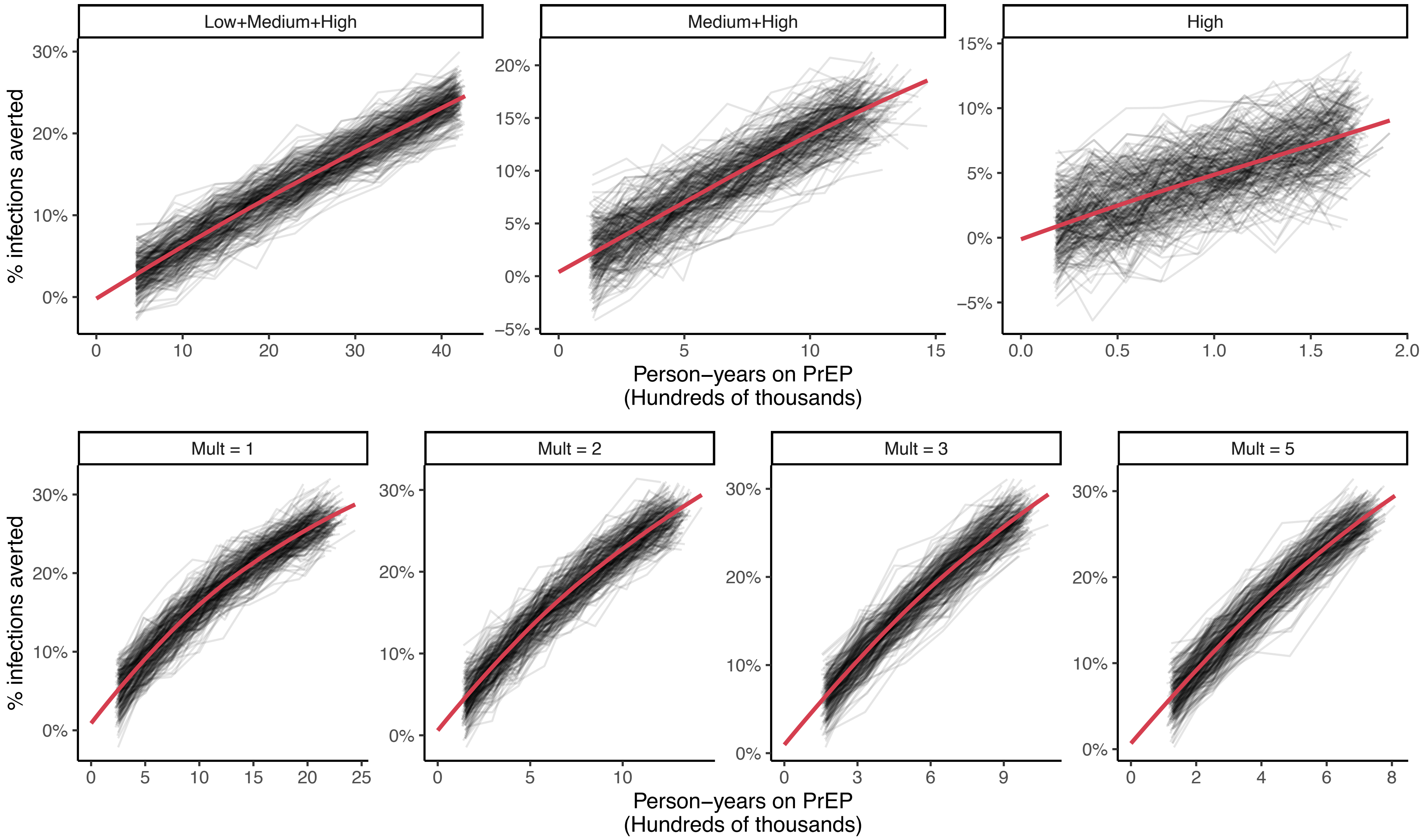

Figure S1.3: Estimation of the additional person-years of PrEP use needed to avert one additional infection relative to a scenario without PrEP (number needed to treat, NNT). Risk Group scenarios are on the top row while the Partnership scenarios are on the bottom row. Each black line represents one of 250 parameter sets. X-axis and y-axis scales vary for each plot to show model fit. The parameter set lines connect simulations run at various PrEP coverage levels (**Tables S1.4** and **S1.5**). The number needed to treat is estimated by fitting a linear model relating person-years on PrEP (as the outcome) to the number of infections averted (as the predictor) with a random effect for parameter set. The estimated coefficient on the number of infections averted predictor indicates the NNT averaged across levels of PrEP coverage.

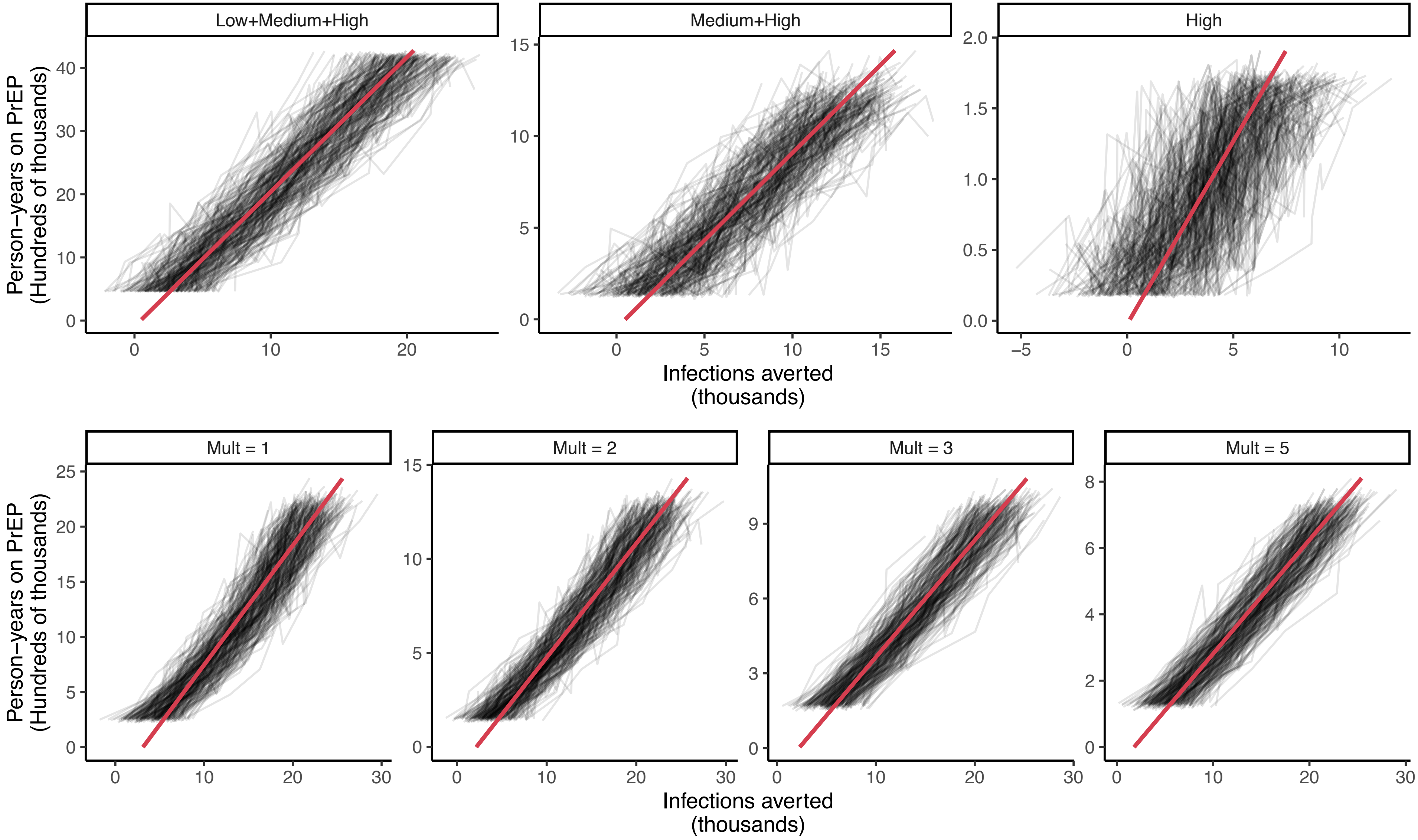

Figure S1.4: Relationship between additional person-time on PrEP and percentage of infections averted relative to a no PrEP scenario, stratified by gender and by PrEP paradigm. Person-years on PrEP and percentage of infections averted are cumulative across a ten-year period spanning 2022 to 2031. Shaded regions indicate 95% credible intervals. Mult = multiplier on the probability that an individual with an HIV-positive partner will use PrEP relative to the probability than an individual with only HIV-negative partners will use PrEP.

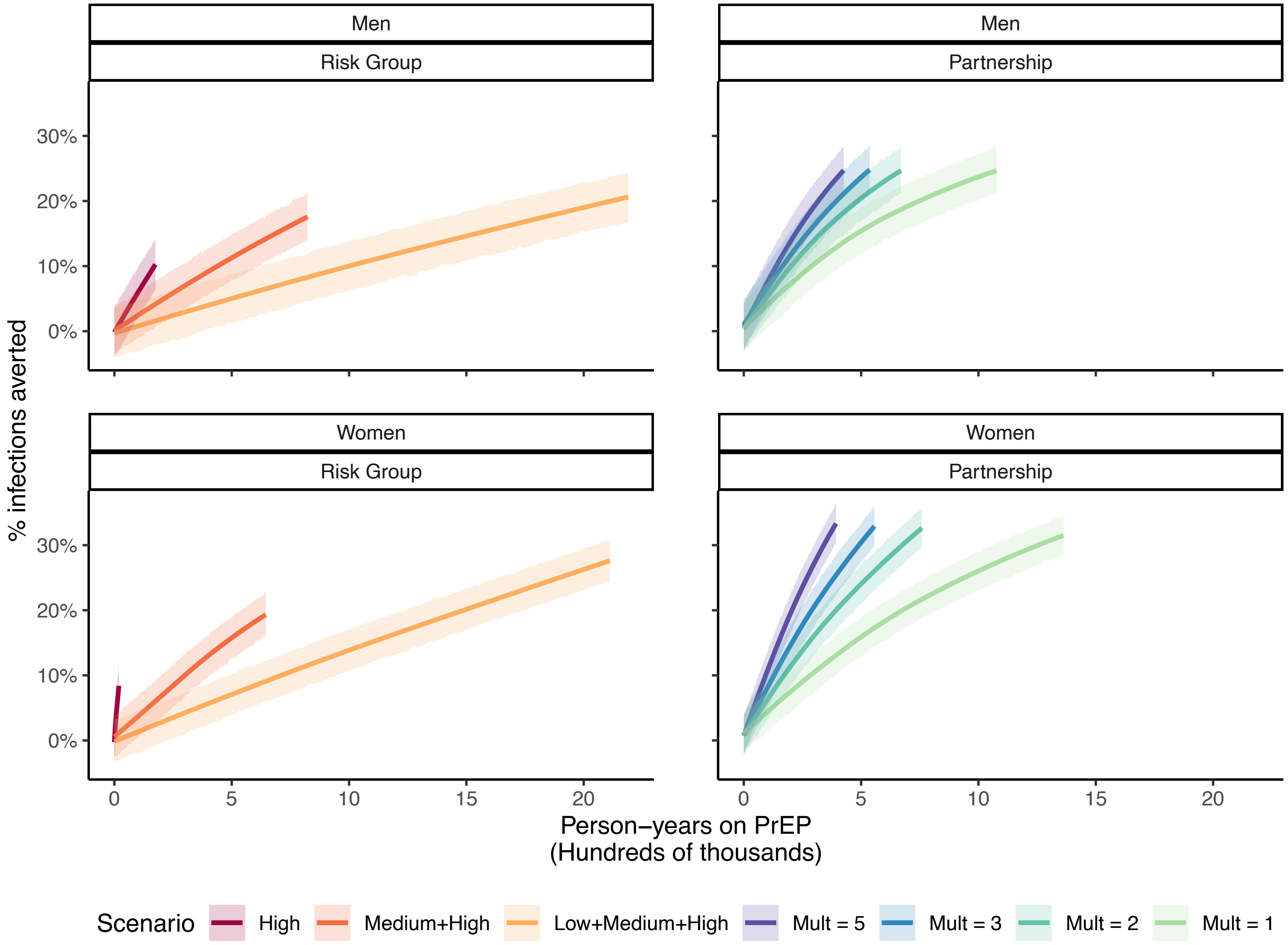

Table S1.6: Number of additional person-years of PrEP needed to avert one additional HIV infection (number needed to treat, NNT), by PrEP scenario and by gender. CI = credible interval. For estimation details, see **Figure S1.3**.

Paradigm	Scenario	Men<br>NNT (95% CI)	Women<br>NNT (95% CI)
Risk Group	All	283 (279-287)	151 (150-152)
	High and medium	118 (116-120)	60 (59-61)
	High	31 (30-32)	3 (3-3)
Partnership	Mult = 1	121 (119-123)	89 (88-90)
	Mult = 2	75 (74-76)	47 (47-48)
	Mult = 3	61 (60-62)	34 (34-35)
	Mult = 5	48 (47-49)	24 (24-24)

Figure S1.5: Relationship between PrEP coverage among all HIV-negative 15–34-year-olds and the relative risk of HIV infection compared to a no PrEP scenario, by PrEP paradigm and by gender. Relative risk is averaged across 2022 to 2031. Shaded regions indicate 95% credible intervals. Mult = multiplier on the probability that an individual with an HIV-positive partner will use PrEP relative to the probability than an individual with only HIV-negative partners will use PrEP.

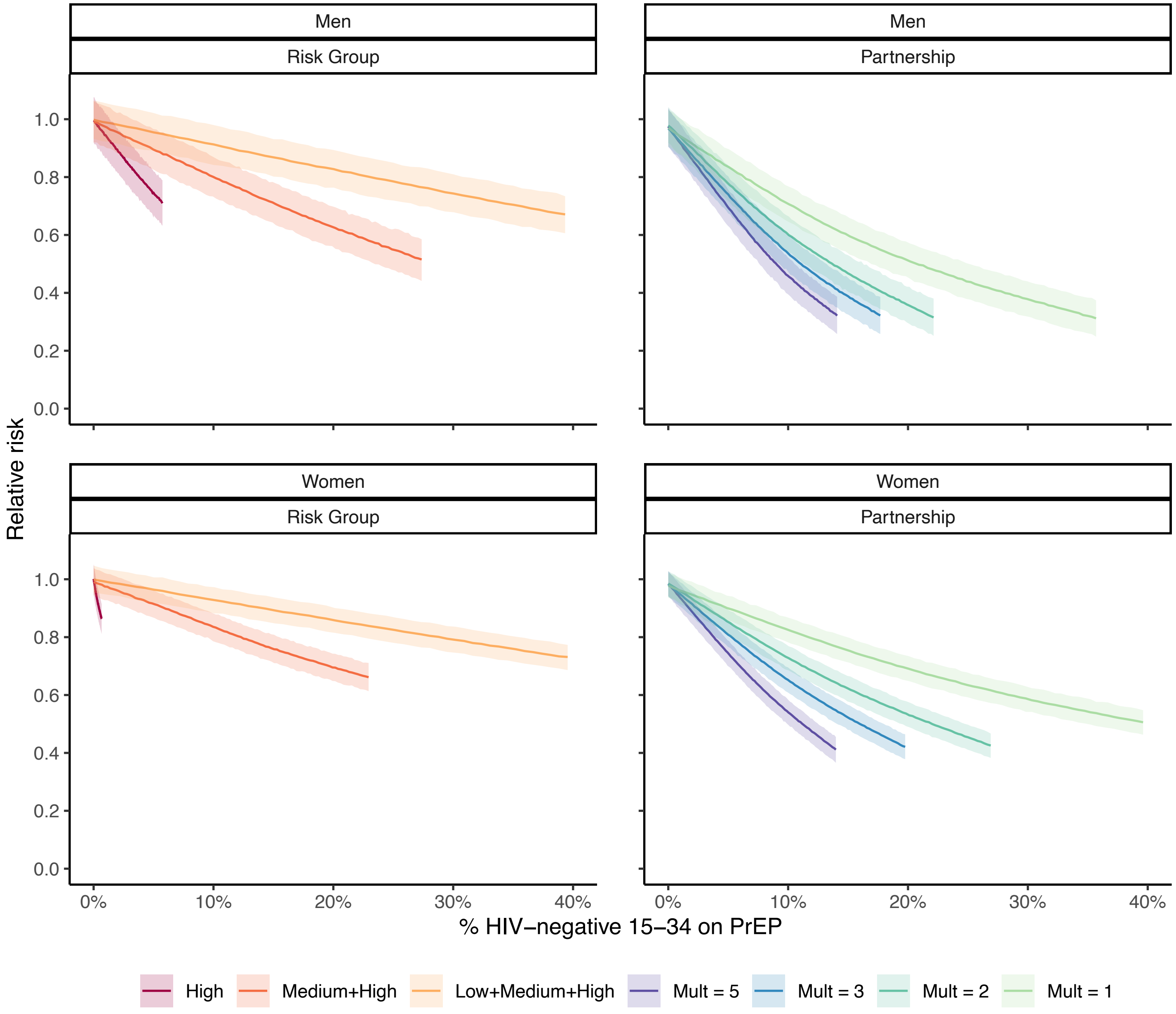

Figure S1.6: Proportion of infections averted between 2022-2031 relative to a no PrEP scenario, disaggregated by risk group of the averted infections and stratified by PrEP scenario. Mult = multiplier on the probability that an individual with an HIV-positive partner will use PrEP relative to the probability than an individual with only HIV-negative partners will use PrEP.

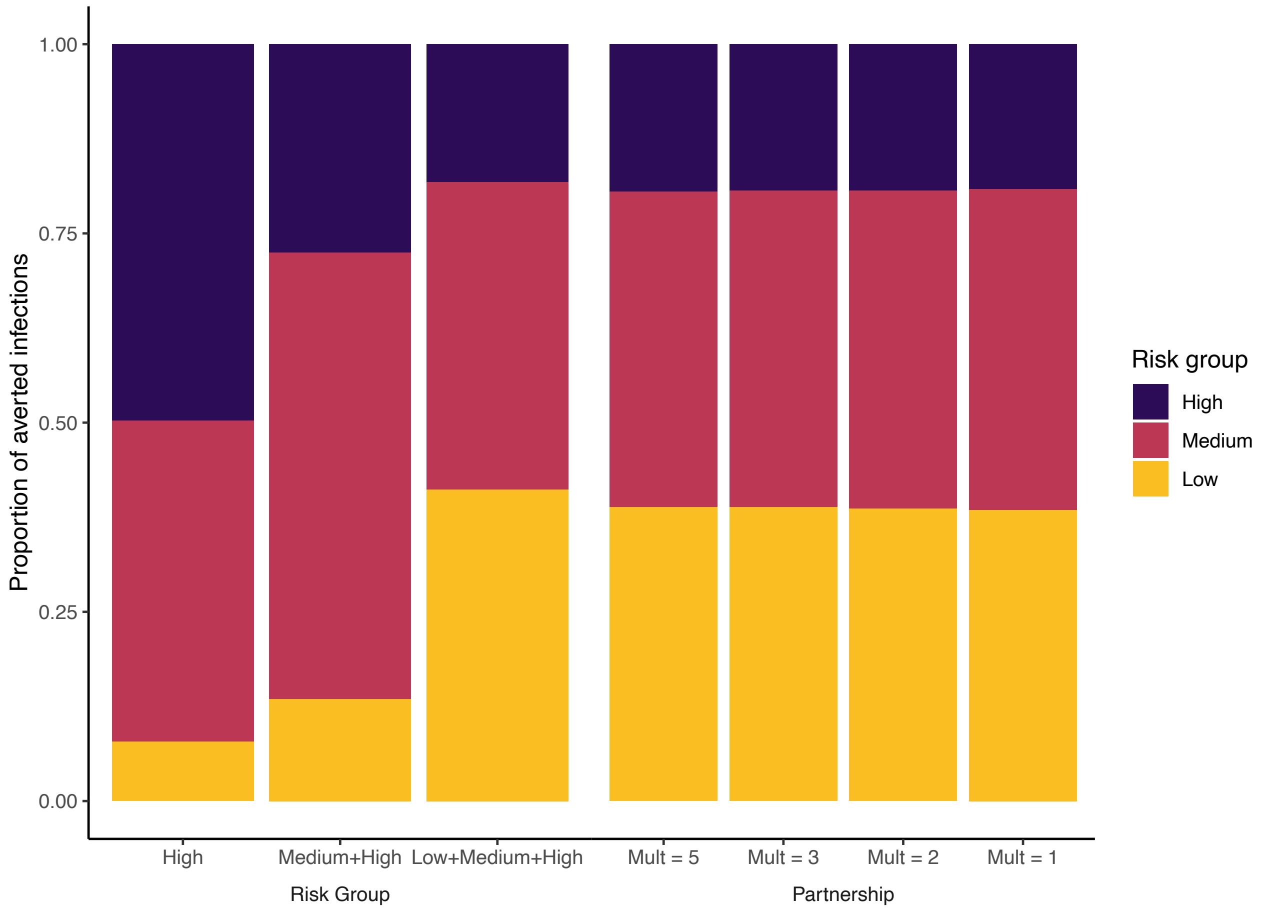

Figure S1.7: Sensitivity analysis of applying the multiplier on PrEP coverage only to individuals with diagnosed HIV-positive partners but not undiagnosed HIV-positive partners. Plot displays the relationship between additional person-time on PrEP and percentage of infections averted relative to a no PrEP scenario in the Partnership paradigm. Solid lines indicate results when the multiplier is applied to individuals with any HIV-positive partner (main analysis), whereas dotted lines indicate that the multiplier is only applied to individuals with an HIV-positive partner who has previously tested positive. Person-years on PrEP and infections averted are cumulative across a ten-year period spanning 2022 to 2031. Shaded regions indicate 95% credible intervals. Mult = multiplier on the probability that an individual with an HIV-positive partner will use PrEP relative to the probability than an individual with only HIV-negative partners will use PrEP.

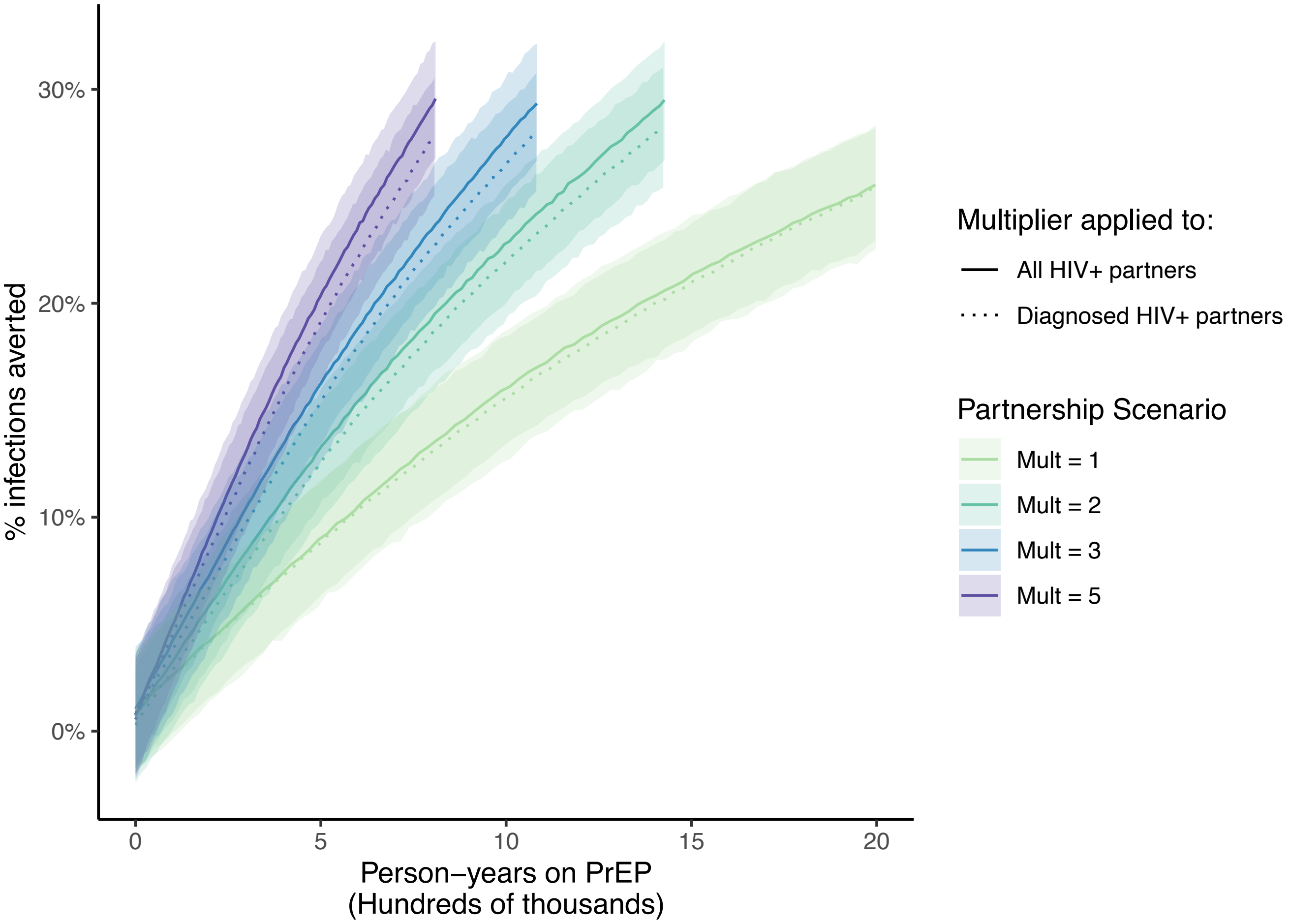

Figure S1.8: Sensitivity of PrEP person-time distribution to restricting the multiplier on PrEP coverage among individuals with HIV-positive partners to require that the partner must have been previously diagnosed. Mult = multiplier on the probability that an individual with an HIV-positive partner will use PrEP relative to the probability than an individual with only HIV-negative partners will use PrEP. Error bars indicate 95% credible intervals across 250 parameter sets.

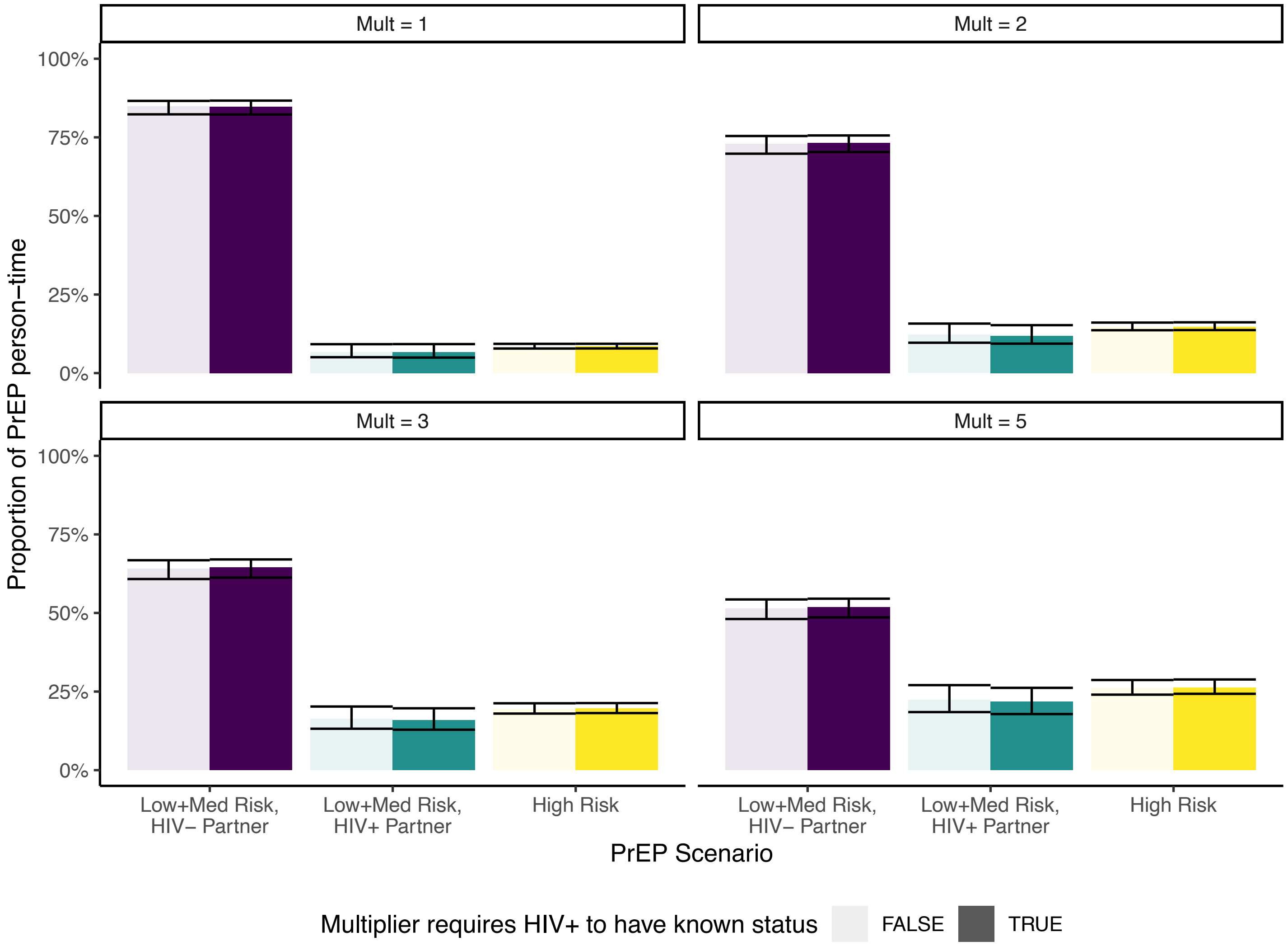

Supplement: Supplementary file 1 — Additional details on risk group sizes, incidence rates and partnership dynamics. [file JIA2-25-e26034-s002.pdf]
