# Supplementary material for: The impact of prevention‐effective PrEP use on HIV incidence: a mathematical modelling study
Source: J Int AIDS Soc. 2022 Nov 17;25(11):e26034. doi: 10.1002/jia2.26034 (PMC9670193; doi:10.1002/jia2.26034)
Supplement: Supplementary file 2 — Model parameters and calibration details. [file JIA2-25-e26034-s001.pdf]

Table S2.1: Select model parameters used to fit the EMOD-HIV transmission model described in [1] to survey data on prevalence, incidence, and ART coverage from the Kingdom of eSwatini. Median and interquartile ranges (IQRs) across 25 best-fitting parameter sets are reported for the 24 dynamic parameters used in the calibration process. A full description of all parameters and references available is at: http://idmod.org/docs/hiv/parameter-configuration.html

Parameter	Description	Dynamic	Static value / fitted median (IQR)	Source
Acute_Duration_In_Months	The time since infection, in months, over which the Acute_Stage_Infectivity_Multiplier is applied to coital acts occurring in that time-period.	no	3	[2]
Acute_Stage_Infectivity_Multiplier	Multiplier acting on Base_Infectivity to determine the per-act transmission probability of an individual during acute stage	no	26	[2]
AIDS_Duration_In_Months	The length of time, in months, prior to an AIDS-related death over which the AIDS_Stage_Infectivity_Multiplier is applied	no	9	[2]
AIDS_Stage_Infectivity_Multiplier	Multiplier acting on Base_Infectivity to determine the per-act transmission probability of an individual during AIDS stage	no	4.5	[2]
ART_CD4_at_Initiation_Saturating_Reduction_in_Mortality	The duration from ART enrollment to on-ART HIV-caused death increases with CD4 at ART initiation up to a threshold determined by this parameter value.	no	350	
ART_dropout	Exponentially distributed mean number of days from ART initiation until ART dropout	no	7300	
ART_Link_Max	The right asymptote for the sigmoid trend of probability of ART linkage (given eligibility) over time.	yes	0.952 (0.948 - 0.955)	
ART_Link_Mid	The time of the inflection point in the sigmoid trend of probability of ART linkage (given eligibility) over time.	yes	2010.7 (2010.4 - 2010.9)	
ART_link_Min	The left asymptote for the sigmoid trend of probability of ART linkage (given eligibility) over time.	no	0	
ART_link_Rate	The slope of the inflection point in the sigmoid trend of probability of ART linkage over time. A Rate of 1 sets the slope to a 25% change in probability per year.	no	1	
ART_Viral_Suppression_Multiplier	Multiplier acting on Base_Infectivity to determine the per-act transmission probability of an individual on ART. Less-than-perfect (<100%) reduction in risk is attributed to sub-optimal adherence, drug resistance, and delay in viral load suppression from ART initiation.	no	0.08	[3]
Base_Infectivity	The probability of transmission when none of the transmission multipliers apply to a coital act (or when all multipliers are set to 1).	yes	0.00233 (0.00231 - 0.00234)	[4]
CD4_At_Death_LogLogistic_Heterogeneity	The inverse shape parameter of a Weibull distribution that represents the at-death CD4 cell count.	no	0.7	
CD4_At_Death_LogLogistic_Scale	The scale parameter of a Weibull distribution hat represents the at-death CD4 cell count.	no	2.96	

CD4_Post_Infection_Weibull_Heterogeneity	The inverse shape parameter of a Weibull distribution that represents the post-acute-infection CD4 cell count.	no	0.2756	
CD4_Post_Infection_Weibull_Scale	The scale parameter of a Weibull distribution that represents the post-acute-infection CD4 cell count.	no	560.43	
Circumcision_Reduced_Acquire	The reduction of susceptibility to HIV by voluntary male medical circumcision (VMMC)	no	0.6	[5–7]
Coital_Act_Rate	Number of coital acts per day for all relationships except commercial ones	no	0.33	
Coital_Act_Rate_Commercial	Number of coital acts per day for commercial relationships	no	0.002739726	
Coital_Dilution_Factor_2_Partners	The multiplicative reduction in the coital act rate for all relationship types when an individual has exactly two current partners. Represents coital dilution.	no	0.75	
Coital_Dilution_Factor_3_Partners	The multiplicative reduction in the coital act rate for all relationship types when an individual has exactly three current partners. Represents coital dilution.	no	0.6	
Coital_Dilution_Factor_4_Plus_Partners	The multiplicative reduction in the coital act rate for all relationship types when an individual has exactly three current partners. Represents coital dilution.	no	0.45	
Commercial_Condom_Max	The maximum asymptote for commercial relationships	no	0.85	
Commercial_Condom_Mid	The year of the inflection point for commercial relationships	no	1999.5	
Commercial_Condom_Min	The minimum asymptote of the probability of condom use per coital act for informal relationships for commercial relationships	no	0.5	
Commercial_Condom_Rate	The rate proportional to the slope at the inflection point for commercial relationships	no	1	
Commercial_Form_Rate	Exponentially distributed mean number new relationships formed per day for commercial relationships	no	0.15	
Condom_Transmission_Blocking_Probability	The per-act multiplier of the transmission probability when a condom is used	no	0.8	
Days_Between_Symptomatic_And_Death_Weibull_Heterogeneity	The time between the onset of AIDS symptoms and death is sampled from a Weibull distribution; this parameter governs the heterogeneity (inverse shape) of the Weibull.	no	0.5	
Days_Between_Symptomatic_And_Death_Weibull_Scale	The time between the onset of AIDS symptoms and death is sampled from a Weibull distribution; this parameter governs the scale of the Weibull.	no	618.34	
Delay_Period_Mean	Delay from HIV infection until ART initiation for future ART scale-up scenarios, post 2016 (in days).	no	180	

HIV_Adult_Survival_Scale_Parameter_Intercept	Determines the intercept of the scale parameter for the Weibull distribution used to determine HIV survival time. Survival time with untreated HIV infection depends on the age of the individual at the time of infection, and is drawn from a Weibull distribution with shape parameter (see HIV_Adult_Survival_Shape_Parameter) and scale parameter. The scale parameter is allowed to vary linearly with age as follows<br>λ = HIV_Adult_Survival_Scale_Parameter_Intercept + HIV_Adult_Survival_Scale_ Parameter_Slope * Age (in years).	no	21.182
HIV_Adult_Survival_Scale_Parameter_Slope	This parameter determines the slope of the scale parameter for the Weibull distribution used to determine HIV survival time.	no	-0.2717
HIV_Adult_Survival_Shape_Parameter	This parameter determines the shape of the Weibull distribution used to determine age-dependent survival time for individuals infected with HIV.	no	2
HIV_Age_Max_for_Adult_Age_Dependent_Survival	Survival time with untreated HIV infection depends on the age of the individual at the time of infection, and is drawn from a Weibull distribution with shape parameter and scale parameters (See HIV_Adult_Survival_Scale_Parameter_Intercept, HIV_Adult_Survival_Scale_ Parameter_Slope, and HIV_Adult_Survival_Shape_Parameter).<br>Although the scale parameter for survival time declines with age, it cannot become negative. To avoid negative survival times at older ages, this parameter, HIV_Age_Max_for_Adult_Age_Dependent_Survival, determines the age beyond which HIV survival is no longer affected by further aging .	no	50
HIV_Age_Max_for_Child_Survival_Function	The maximum age at which an individual's survival will be fit to the child survival function. If the value of this parameter falls between zero and the age of sexual debut, model results are not sensitive to this parameter as there is no mechanism for children to become infected between infancy and sexual debut.	no	15
HIV_Child_Survival_Rapid_Progressor_Fraction	The proportion of HIV-infected children who are rapid HIV progressors.	no	0.57
HIV_Child_Survival_Rapid_Progressor_Rate	The exponential decay rate, in years, describing the distribution of HIV survival for children who are rapid progressors.	no	1.52
HIV_Child_Survival_Slow_Progressor_Scale	The Weibull scale parameter describing the distribution of HIV survival for children who are slower progressors.	no	16
HIV_Child_Survival_Slow_Progressor_Shape	The Weibull shape parameter describing the distribution of HIV survival for children who are slower progressors.	no	2.7
Informal_Condom_Max	The maximum asymptote for informal relationships	yes	0.337 (0.321 - 0.355)
Informal_Condom_Mid	The year of the inflection point for informal relationships	yes	1992.6 (1992.2 - 1992.9)

Informal_Condom_Min	The minimum asymptote of the probability of condom use per coital act for informal relationships	no	0
Informal_Condom_Rate	The rate proportional to the slope at the inflection point for informal relationships		3.003 (2.941 - 3.076)
Informal_Form_Rate	Exponentially distributed mean number new relationships formed per day for informal relationships	yes	0.00146 (0.00134 - 0.00155)
Male_To_Female_Relative_Infectivity_Multiplier_Old	An array of scale factors governing the susceptibility of females relative to males, by age ≥ 25	yes	2.844 (2.727 - 2.958)
Male_To_Female_Relative_Infectivity_Multiplier_Young	An array of scale factors governing the susceptibility of females relative to males, by age < 25	yes	4.894 (4.747 - 5.041)
Marital_Condom_Max	The maximum asymptote for marital relationships	yes	0.218 (0.207 - 0.231)
Marital_Condom_Mid	The year of the inflection point for marital relationships	yes	2001.8 (2001.5 - 2002.1)
Marital_Condom_Min	The minimum asymptote of the probability of condom use per coital act for informal relationships for marital relationships	no	0
Marital_Condom_Rate	The rate proportional to the slope at the inflection point for marital relationships	yes	2.407 (2.252 - 2.524)
Marital_Form_Rate	Exponentially distributed mean number new relationships formed per day for marital relationships	yes	0.00046 (0.00044 - 0.0005)
Maternal_Infection_Transmission_Probability	The probability of transmission of infection from mother to infant at birth.		0.3
Maternal_Transmission_ART_Multiplier	The maternal transmission multiplier for on-ART mothers.	no	0.03334
preART_Link_Max	The right asymptote for the sigmoid trend of probability of preART linkage (given eligibility) over time.	yes	0.807 (0.783 - 0.829)
preART_Link_Mid	The time of the inflection point in the sigmoid trend of probability of preART linkage (given eligibility) over time.	yes	1995.7 (1995.1 - 1996.4)
preART_link_Min	The left asymptote for the sigmoid trend of probability of preART linkage (given eligibility) over time.	yes	0.00325 (0 - 0.03031)
preART_link_Rate	The slope of the inflection point in the sigmoid trend of probability of preART linkage over time. A Rate of 1 sets the slope to a 25% change in probability per year.	no	1
Proportion_Low_Risk	Proportion of the intial population that is low risk	yes	0.73 (0.721 - 0.742)
Seed_Year	Year in which the epidemic is seeded into high risk groups	yes	1982.7 (1982.4 - 1983.2)
Sexual_Debut_Age_Female_Weibull_Heterogeneity	The inverse shape of the Weibull distribution for female debut age.	yes	0.309 (0.293 - 0.322)
Sexual_Debut_Age_Female_Weibull_Scale	The scale term of the Weibull distribution for female debut age.	yes	16.302 (16.166 - 16.396)
Sexual_Debut_Age_Male_Weibull_Heterogeneity	The inverse shape of the Weibull distribution for male debut age.	yes	0.042 (0.04 - 0.05)
Sexual_Debut_Age_Male_Weibull_Scale	The scale term of the Weibull distribution for male debut age.	yes	17.499 (17.357 - 17.699)

Sexual_Debut_Age_Min	The minimum age at which individuals become eligible to form sexual relationships.	no	13
Transitory_Condom_Max	The maximum asymptote for transitory relationships	yes	0.103 (0.089 - 0.117)
Transitory_Condom_Mid	The year of the inflection point for transitory relationships	yes	1996.7 (1996.1 - 1997)
Transitory_Condom_Min	The minimum asymptote of the probability of condom use per coital act for informal relationships for transitory relationships	no	0
Transitory_Condom_Rate	The rate proportional to the slope at the inflection point for transitory relationships	yes	2.998 (2.878 - 3.106)
Transitory_Form_Rate	Exponentially distributed mean number new relationships formed per day for transitory relationships	no	0.001047839
Transitory_Weibull_Heterogeneity	Inverse of the Weibull shape (1/kappa) parameter of relationship duration in years for transitory relationships	no	0.833333333
Transitory_Weibull_Scale	Weibull scale parameter of relationship duration in years for transitory relationships.	no	0.956774771

Figure S2.1: Model HIV incidence rates per 100 person-years (colored lines) and 95% credible intervals (shaded regions) by gender among adults ages 15-49 compared to observed data (points and 95% confidence interval error bars). SHIMS = Swaziland HIV Incidence Measurement Survey

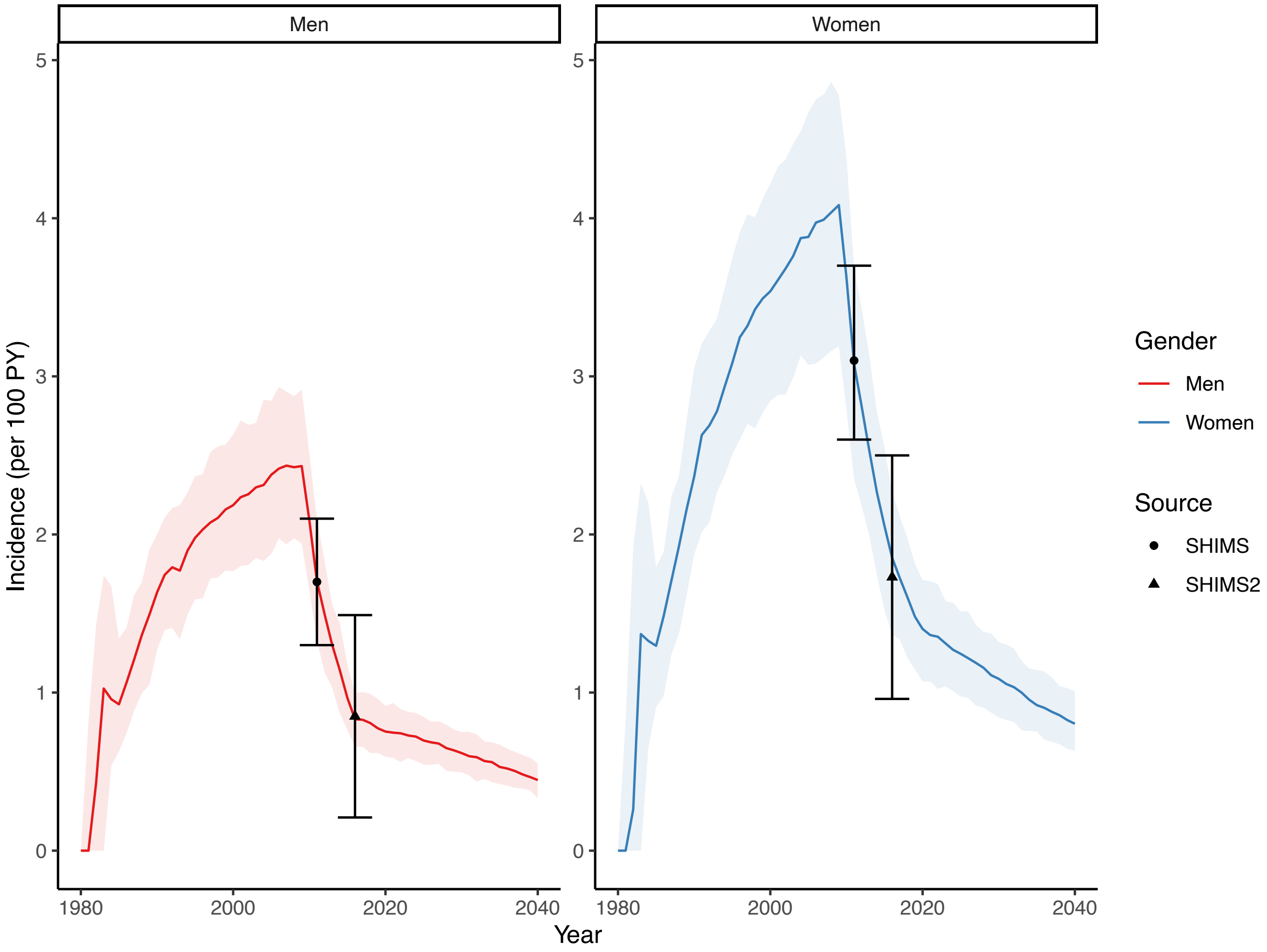

Figure S2.2: Model HIV prevalence (colored lines) and 95% credible intervals (shaded regions) by gender and five-year age group compared to observed data (points and 95% confidence interval error bars). DHS = Demographic and Health Survey; SHIMS = Swaziland HIV Incidence Measurement Survey

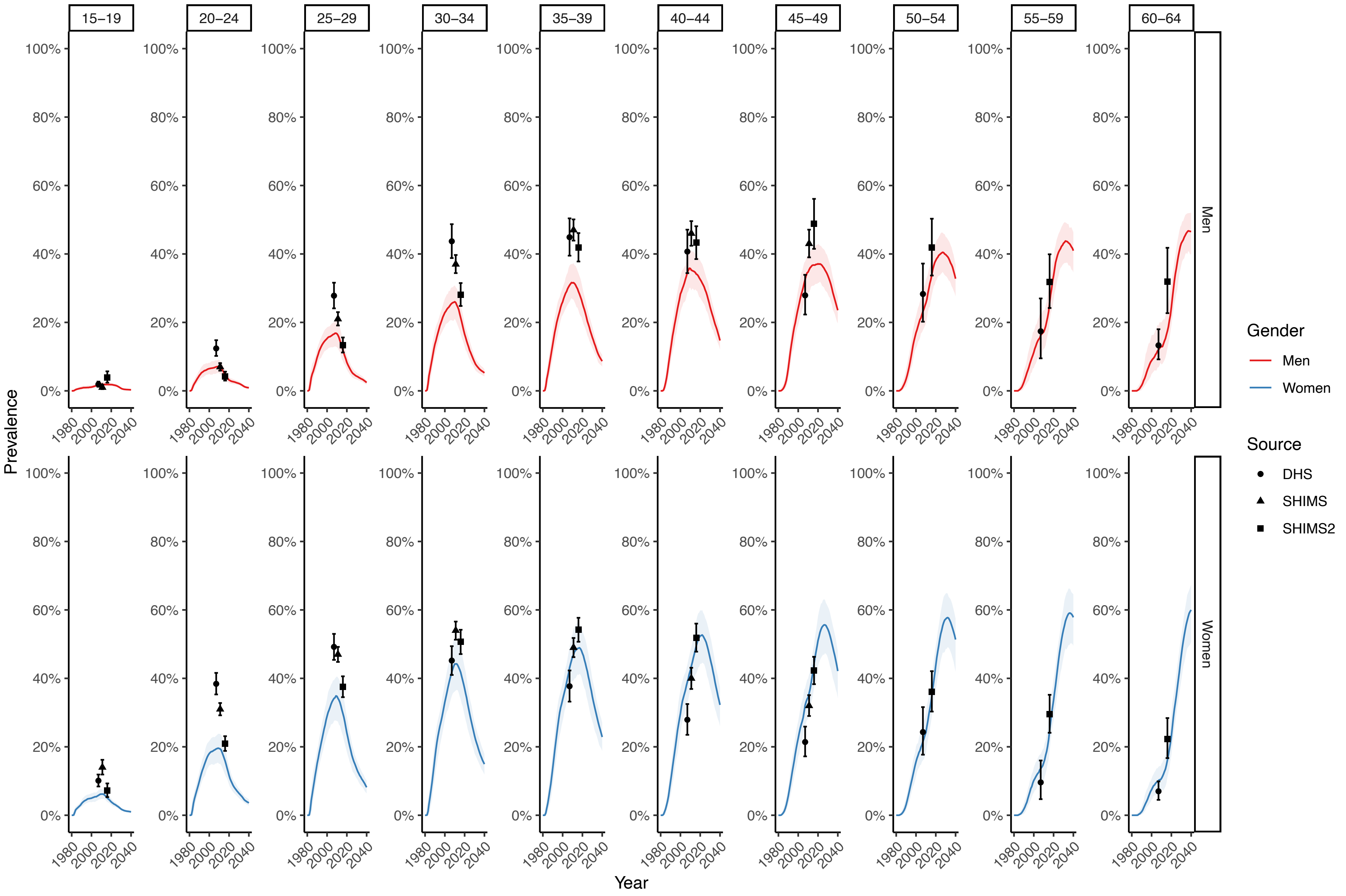

Figure S2.3: Model ART coverage (colored lines) and 95% credible intervals (shaded regions) by gender, for adults ages 15-49. Dashed lines indicate UNAIDS ART coverage target (90% of HIV+ knowing status * 90% of HIV+ with known status on ART = 81% ART coverage among HIV+) by the year 2020.

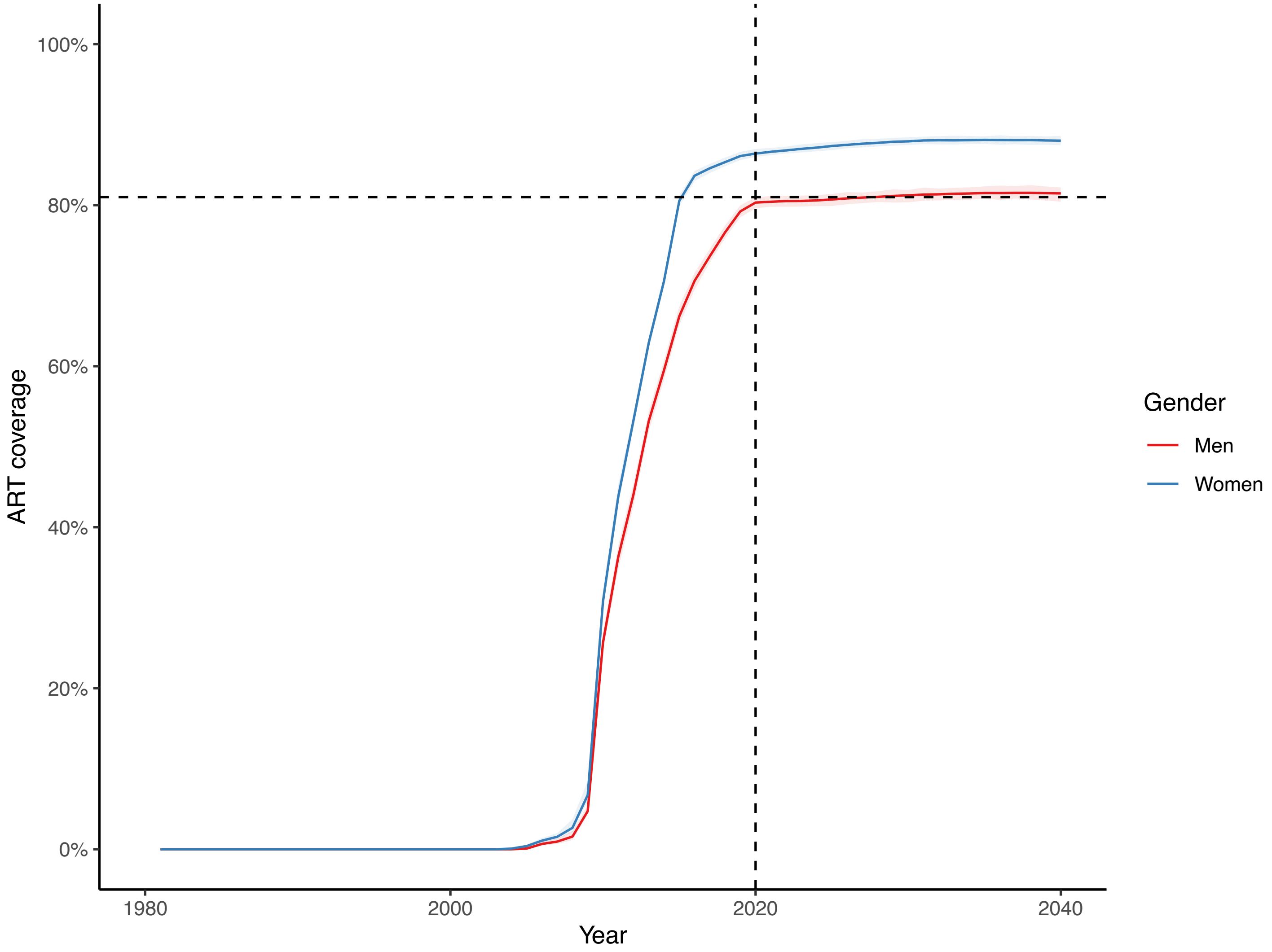

References

1. Akullian A, Morrison M, Garnett GP, Mnisi Z, Lukhele N, Bridenbecker D, et al. The effect of 90-90-90 on HIV-1 incidence and mortality in eSwatini: a mathematical modelling study. Lancet HIV. 2020 Feb;7(5):e348–58.

2. Hollingsworth TD, Anderson RM, Fraser C. HIV-1 Transmission, by Stage of Infection. J Infect Dis. 2008 Sep;198(5):687–93.

3. Donnell D, Baeten JM, Kiarie J, Thomas KK, Stevens W, Cohen CR, et al. Heterosexual HIV-1 transmission after initiation of antiretroviral therapy: a prospective cohort analysis. The Lancet. 2010 Jun;375(9731):2092–8.

4. Wawer MJ, Gray RH, Sewankambo NK, Serwadda D, Li X, Laeyendecker O, et al. Rates of HIV-1 transmission per coital act, by stage of HIV-1 infection, in Rakai, Uganda. J Infect Dis. 2005 May 1;191(9):1403–9.

5. Auvert B, Taljaard D, Lagarde E, Sobngwi-Tambekou J, Sitta R, Puren A. Randomized, controlled intervention trial of male circumcision for reduction of HIV infection risk: The ANRS 1265 trial. PLoS Med. 2005;2(11):1112–22.

6. Bailey RC, Moses S, Parker CB, Agot K, Maclean I, Krieger JN, et al. Male circumcision for HIV prevention in young men in Kisumu, Kenya: a randomised controlled trial. Lancet. 2007 Feb;369(9562):643–56.

7. Gray RH, Kigozi G, Serwadda D, Makumbi F, Watya S, Nalugoda F, et al. Male circumcision for HIV prevention in men in Rakai, Uganda: a randomised trial. Lancet. 2007 Feb;369(9562):657–66.
